# Berberrubine inhibits *Helicobacter pylori* by inducing oxidative stress and impairing membrane integrity

**DOI:** 10.1002/mlf2.70061

**Published:** 2026-02-19

**Authors:** Minzhi Jiang, Changyu Wang, Kai Wang, Xinchi Feng, Gen Li, Yu Jiang, Xue Wang, Shijie Cao, Liqin Ding, Shuangyu Bi, Feng Qiu, Shuang‐Jiang Liu, Chang Liu

**Affiliations:** ^1^ State Key Laboratory of Microbial Technology Shandong University Qingdao China; ^2^ Division of Life Sciences and Medicine University of Science and Technology of China Hefei China; ^3^ School of Chinese Materia Medica, and Tianjin Key Laboratory of Therapeutic Substance of Traditional Chinese Medicine Tianjin University of Traditional Chinese Medicine Tianjin China; ^4^ State Key Laboratory of Component‐based Chinese Medicine Tianjin University of Traditional Chinese Medicine Tianjin China; ^5^ State Key Laboratory of Microbial Resources, and Environmental Microbiology Research Center (EMRC), Institute of Microbiology Chinese Academy of Sciences Beijing China; ^6^ University of Chinese Academy of Sciences Beijing China

## Abstract

*Helicobacter pylori* is a major gastric pathogen with increasing antibiotic resistance, creating an urgent need for new therapeutic strategies. We screened 37 pure compounds and 9 herbal extracts for anti‐*H. pylori* activity and identified berberrubine as the most potent agent, with a minimum inhibitory concentration of 11 μg/ml. Berberrubine exhibited bacteriostatic effects by inducing oxidative stress and disrupting membrane integrity, as demonstrated by transcriptomic analysis, reactive oxygen species (ROS) accumulation, and structural damage, all of which were alleviated by the antioxidant N‐acetylcysteine. Similar inhibitory effects were observed in *Escherichia coli*, indicating broader antimicrobial potential. This study provides the mechanistic evidence of berberrubine's activity against *H. pylori*, highlighting its promise as a candidate for development into alternative therapies to address antibiotic resistance.

Increasing evidence indicates that gastrointestinal microbes play critical roles in cancer development^1^, ^2^, ^3^, ^4^, ^5^, with *Helicobacter pylori* being one of the most strongly associated pathogens in gastric carcinogenesis^4^, ^6^. In the absence of effective drug intervention, *H. pylori* can persist in the human body for decades, contributing to chronic inflammation and cellular damage. The persistence of *H. pylori* is largely attributed to its remarkable resistance to various antibiotics, posing a significant challenge to its effective eradication. The most commonly used treatments for *H. pylori* infections in clinical settings are quadruple therapies. These regimens are either bismuth‐based, involving a combination of bismuth (a topical agent), tetracycline, metronidazole, and a proton pump inhibitor (PPI), or non‐bismuth quadruple therapy, also known as concomitant therapy^7^, ^8^. The latter combines a PPI with amoxicillin, clarithromycin, and metronidazole, administered simultaneously rather than sequentially^9^. Although these strategies largely improve treatment efficacy and reduce the risk of antibiotic resistance, challenges persist due to the growing prevalence of drug resistance and frequent treatment failures. Therefore, it is imperative to explore more effective bacteriostatic strategies against *H. pylori* as alternatives or complements to existing antibiotic therapies.

An increasing number of research studies have demonstrated that plant‐derived natural compounds exhibit inhibitory effects against pathogens, including *H. pylori* ^10^, ^11^, ^12^, ^13^. Plant‐derived natural compounds have emerged as promising candidates, with crude extracts showing inhibitory activity against *H. pylori* but limited by their complexity and uncertain pharmacokinetics. Among them, the isoquinoline alkaloid berberine, isolated from *Coptis chinensis* and *Phellodendron amurense*, is the most extensively studied^14^, ^15^, ^16^. These promising results suggest that plant‐derived natural products could be a treasure trove of resources for discovering antibiotic alternatives, warranting further exploration.

In this study, from our natural product library, we screened 37 pure compounds (purity >95%, chemical structures shown in Figure S1), 3 ethanol extracts, and 6 aqueous extracts from medicinal herbs, and determined their minimum inhibitory concentrations (MICs) against *H. pylori in vitro*. As shown in Table S1, 15 compounds from 4 herbs exhibited antibacterial activity, while the other 31 showed no activity. Notably, all 6 purified compounds and aqueous extracts derived from *Coptidis rhizoma* demonstrated inhibitory effects against *H. pylori*, with berberrubine showing the strongest antibacterial activity, achieving a MIC of 11 μg/ml. Additionally, two other compounds derived from *C. rhizoma*, epiberberine and berberine, exhibited the second and third strongest antibacterial effects, with MIC of 12.5 and 25 μg/ml, respectively. Structure–activity relationship (SAR) analysis of berberine derivatives revealed that antibacterial activity depended on the protoberberine core and was influenced by the type and position of substituents (Figure S2). At the C2 position, a methylenedioxy group conferred a higher activity (e.g., berberine), outperforming methoxy (e.g., palmatine) and hydroxyl (e.g., columbamine). In contrast, at the C9 position, a hydroxyl group (e.g., berberrubine) showed greater potency than methylenedioxy (e.g., epiberberine), following the order: hydroxyl > methylenedioxy > methoxy. Notably, more substituents did not necessarily improve activity, e.g., coptisine, which has two methylenedioxy groups, did not exhibit enhanced efficacy.

Furthermore, to determine whether berberrubine exhibited bactericidal or bacteriostatic activity against *H. pylori*, we measured its minimum bactericidal concentration (MBC). Based on the previously established MIC, berberrubine was tested at final concentrations of 11, 22, 44, 88, 176, and 352 μg/ml (Figure S3). The results indicated an MBC of 176 μg/ml, corresponding to an MBC/MIC ratio of 16, suggesting that berberrubine primarily exhibited bacteriostatic activity against *H. pylori*. According to established criteria^17^, ^18^, this MBC/MIC ratio indicated that berberrubine primarily exhibited a bacteriostatic rather than a bactericidal effect.

To elucidate the bacteriostatic mechanism underpinned by the observed inhibitory activity, *H. pylori* cells were treated with berberrubine (2× MIC group) and without (Control group) for 2 h, respectively. RNA‐seq‐based transcriptomic analysis was conducted on bacterial cells at the initial time point (Control 0 and 2× MIC 0 group) and after 2 h treatment (Control 1 and 2× MIC 1 group) to compare differential gene expression. The Kendall correlation coefficients were employed to assess the similarity in transcriptional profiles between any two datasets, which indicated that, in addition to drug treatment, cultivation time also affected gene expression (Figure S4). The Principal Component Analysis (PCA) further revealed significant changes in the transcriptional levels between the Control 0 to Control 1 and 2× MIC 0 to 2× MIC 1 samples (Figure 1A), which corroborated the influence of cultivation time on gene expression. These results indicated that when analyzing differentially expressed genes (DEGs) caused by drug treatment, the transcriptional changes with time should be excluded.

**Figure 1:**
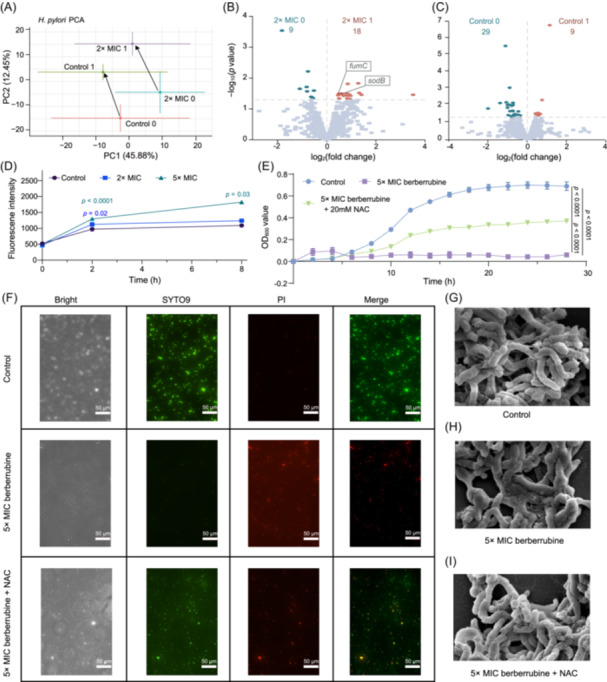
Transcriptome analysis of *Helicobacter pylori* after 2 h berberrubine exposure and evaluation of intracellular ROS and membrane integrity under 2× MIC and 5× MIC treatments. (A) Principal Component Analysis (PCA) showing transcriptional differences across time points and treatments (Control 0 vs. Control 1; 2× MIC 0 vs. 2× MIC 1). (B) Volcano plot of DEGs in 2× MIC 0 vs. 2× MIC 1 comparison (|log_2_ FC | > 0, *p* < 0.05). (C) Volcano plot of DEGs in Control 0 vs. Control 1 comparison (|log_2_ FC | >0, *p* < 0.05). (D) Quantification of intracellular ROS levels in *H. pylori* following treatment with vehicle control or berberrubine (2× and 5× MIC). (E) Growth curves of *H. pylori* following treatment with vehicle control, 5× MIC berberrubine, or berberrubine in combination with 20 mM NAC. (F) Images of *H. pylori* cells stained with the BacLight™ bacterial viability kit. SYTO 9 (green) indicates live cells, while PI (red) indicates dead cells. Scale bar, 50 µm. (G–I) SEM images showing *H. pylori* cell morphology after 2 h treatment of vehicle control (G), 5× MIC berberrubine (H), and co‐treatment of 5× MIC berberrubine and 20 mM NAC (I). Scale bar, 1 µm. Data are shown as the mean ± S.E.M. The *p* value was calculated by Student's *t*‐test and labeled at the corresponding data point. DEGs, differentially expressed genes; MIC, minimum inhibitory concentration; NAC, N‐Acetylcysteine; PI, propidium iodide; ROS, reactive oxygen species.

DEG analysis revealed that berberrubine treatment induced distinct transcriptional responses in *H. pylori*. When comparing berberrubine‐treated samples (2× MIC 1) with their baseline counterparts (2× MIC 0), 18 genes were significantly upregulated, among which 14 appeared to be specifically induced by the compound rather than by time‐dependent effects (Figure 1B). Functional annotation revealed that several of these genes were associated with oxidative stress responses, including *sodB* (superoxide dismutase), *fumC* (class II fumarate hydratase), and *OW489_RS07460* (a pyruvate:flavodoxin oxidoreductase). However, these genes did not exhibit significant differential expression when compared between Control 0 and Control 1 group (Figure 1C). Additionally, redox‐active components such as *Novel00152* (cytochrome‐like protein) and *Novel00227* (iron/manganese superoxide dismutase homolog) were also upregulated, suggesting enhanced activation of oxidative stress pathways (Table S2). Genes related to membrane structure and repair were concurrently elevated, including *pgsA* (phosphatidyltransferase), *hofD* (outer membrane beta‐barrel protein), and *OW489_RS06350* (branched‐chain amino acid transporter permease), potentially reflecting membrane perturbation or compensatory repair mechanisms. Gene ontology (GO) enrichment analysis supported these findings. While control samples (Control 1 vs. Control 0) primarily exhibited enrichment in cell proliferation and nucleoside binding, berberrubine‐treated samples showed significant enrichment in oxidation–reduction processes, energy metabolism, respiration, and drug metabolism (Figure S5A,B).

To further characterize drug‐specific effects, we also compared berberrubine‐treated samples (2× MIC 1) with time‐matched untreated controls (Control 1) and identified 34 uniquely upregulated genes after exposure (Figure S5C,D). Among these, several were implicated in redox regulation and oxidative stress, including *Novel00101* (flavodoxin‐like oxidoreductase), *Novel00019* (aldehyde dehydrogenase), *OW489_RS05840* (alkyl hydroperoxide reductase), and NADH‐ubiquinone oxidoreductase subunits *Novel00183* and *Novel00186*, consistent with enhanced redox cycling and oxidative burden. In parallel, upregulation of genes linked to membrane integrity, such as *Novel00119* (ompA‐family protein), *hofD*, *Novel00145* (TonB‐dependent receptor domain), *pgsA*, and urease‐related genes, including *ureA* and *Novel00023*, possibly reflecting membrane perturbation. Additional activation of genes involved in translation and energy metabolism, including ribosomal proteins (*Novel00146*, *Novel00171*, *Novel00173*, and *rpsQ*) and ATP synthase subunit (*Novel00215*), further indicated increased energetic and biosynthetic demand during adaptive stress responses. These findings were further supported by GO enrichment analysis (Figure S5E), which revealed significant enrichment of biological processes, such as cellular amide and organonitrogen compound biosynthesis, as well as molecular functions including structural molecule activity, lipid/phospholipid binding, and transition metal ion binding.

Above comparisons of DEGs highlighted a consistent transcriptional signature involving oxidative stress and membrane‐related pathways, providing molecular clues that such processes might be associated with the bacteriostatic activity of berberrubine.

To experimentally validate the transcriptomic indications of oxidative stress and membrane impairment, we assessed the physiological effects of berberrubine treatment in *H. pylori*. Intracellular reactive oxygen species (ROS) levels were quantified by 2′,7′‐dichlorofluorescein diacetate (DCFH‐DA) staining. Treatment with 2× and 5× MIC concentrations of berberrubine led to a rapid increase in reactive oxygen species (ROS) accumulation within 2 h, with sustained elevation observed in the 5× MIC group after 8 h, indicating a dose‐dependent induction of oxidative stress (Figure 1D). To determine whether this oxidative stress contributes directly to the bacteriostatic activity of berberrubine, bacterial growth was evaluated in the presence or absence of the antioxidant N‐acetylcysteine (NAC). While 5× MIC berberrubine completely inhibited growth, co‐treatment with 20 mM NAC significantly restored viability (Figure 1E), suggesting that disruption of redox homeostasis played a causal role in the growth‐inhibitory effect of berberrubine.

We next evaluated membrane integrity using SYTO9/PI dual‐fluorescence staining at 2 h posttreatment. Untreated control cells displayed strong SYTO9 (green) fluorescence, indicative of intact membranes and viable cells. In contrast, berberrubine‐treated cells exhibited a significant increase in propidium iodide (PI) (red) fluorescence, reflecting compromised membrane integrity and loss of viability. Co‐treatment with NAC significantly reduced PI‐positive cell proportions, demonstrating a protective effect against membrane damage (Figure 1F). Scanning electron microscopy (SEM) further corroborated these findings. Control cells exhibited smooth, flat membrane surfaces with a full, plump morphology and evenly distributed cytoplasm (Figure 1G), whereas berberrubine‐treated cells showed widespread surface wrinkling, membrane collapse, and cytoplasmic leakage (Figure 1H). NAC co‐treatment visibly attenuated these changes, preserving general cell morphology, although mild surface wrinkling and reduced cell fullness persisted (Figure 1I).

These results provided experimental evidence supporting transcriptomic predictions that berberrubine disrupted redox homeostasis and compromised membrane integrity in *H. pylori*. The alleviating effects of NAC further highlighted oxidative stress as a central contributor to the bacteriostatic activity of bacteriostatic.

To validate the universality of berberrubine's antibacterial effect, *Escherichia coli*, a representative intestinal opportunistic pathogen, was selected to evaluate its sensitivity to berberrubine and the other 45 natural products previously screened for *H. pylori*. As shown in Table S3, 12 compounds from 5 herbs exhibited antibacterial activity. Similar to *H. pylori*, *E. coli* was sensitive to all 5 purified compounds and aqueous extracts derived from *C. rhizoma*, with berberrubine showing the strongest antibacterial effect. The MIC of berberrubine against *E. coli* was 170 μg/ml.

Furthermore, ROS accumulation was assessed using DCFH‐DA after treatment with 2× MIC and 5× MIC concentrations of berberrubine. After 2 h treatment, significant increases in intracellular ROS levels were observed in both 2× MIC and 5× MIC treatment groups compared to the control group (Figure S6A). Similar to the effect in *H. pylori*, ROS accumulation in *E. coli* was dose‐dependent and persisted until 8 h. Further SEM images revealed that, in contrast to the structurally intact cells in the control group, berberrubine‐treated *E. coli* exhibited membrane wrinkling of the cell membrane and cellular damage, with these morphological changes becoming more severe as berberrubine concentrations increased (Figure S6B–D). These results indicated that, similar to *H. pylori*, berberrubine caused oxidative stress and membrane damage in *E. coli*, thereby exerting its inhibitory effect.

In summary, we identified berberrubine as a potent antibacterial candidate with bacteriostatic effects against *H. pylori* and *E. coli*. Its mechanism primarily involves the induction of oxidative stress and disruption of membrane integrity. ROS‐induced damage to cellular components, such as membranes and DNA, is a well‐known antimicrobial mechanism^19^, ^20^. Co‐treatment with NAC alleviated berberrubine‐induced oxidative stress and partially restored bacterial growth and membrane stability, but residual inhibition remained, suggesting that oxidative stress is not the sole mechanism. Berberrubine may act through additional pathways, such as direct membrane disruption or interference with other cellular processes. The similarity of its effects in *E. coli* suggests broad‐spectrum potential, positioning berberrubine as a promising candidate for managing diverse bacterial infections, particularly in the gastrointestinal tract. However, further studies are needed to evaluate its pharmacokinetics, bioavailability, and toxicity, as well as to validate its effectiveness *in vivo*. Overall, berberrubine represents a promising lead compound for antimicrobial development in the context of rising antibiotic resistance.

## ETHICS STATEMENT

The authors have nothing to report.

## Supporting information

Supplementary figures251128.

Supplementary method.

Supplementary method.

## Data Availability

Data are deposited in the National Microbiology Data Center (NMDC) with accession number NMDC10019606 (https://nmdc.cn/resource/genomics/project/detail/NMDC10019606).
