# Supplementary material for: Berberrubine inhibits Helicobacter pylori by inducing oxidative stress and impairing membrane integrity
Source: mLife. 2026 Feb 19;5(1):126–30. doi: 10.1002/mlf2.70061 (PMC12948479; doi:10.1002/mlf2.70061)
Supplement: Supplementary file 1 — Supplementary figures251128. [file MLF2-5-126-s001.docx]

**Berberrubine inhibits *Helicobacter pylori* by inducing oxidative stress and impairing membrane integrity**

Min-Zhi Jiang, Chang-Yu Wang, Kai Wang, Xin-Chi Feng, Gen Li, Yu Jiang, Xue Wang, Shi-Jie Cao, Li-Qin Ding, Shuang-Yu Bi, Feng Qiu*, Shuang-Jiang Liu*, Chang Liu*

Min-Zhi Jiang: State Key Laboratory of Microbial Technology, Shandong University, Qingdao 266000, P. R. China. [jiangminzhi2006@126.com](mailto:jiangminzhi2006@126.com)

Chang-Yu Wang: Division of Life Sciences and Medicine, University of Science and Technology of China, 230000, P. R. China. [wcy0817@mail.ustc.edu.cn](mailto:wcy0817@mail.ustc.edu.cn)

Kai Wang: School of Chinese Materia Medica, and Tianjin Key Laboratory of Therapeutic Substance of Traditional Chinese Medicine, Tianjin University of Traditional Chinese Medicine, Tianjin, 301617, China. [kaiwang_2009@163.com](mailto:kaiwang_2009@163.com)

Xin-Chi Feng: School of Chinese Materia Medica, and Tianjin Key Laboratory of Therapeutic Substance of Traditional Chinese Medicine, Tianjin University of Traditional Chinese Medicine, Tianjin, 301617, China. [xiaochi0211@163.com](mailto:xiaochi0211@163.com)

Gen Li: School of Chinese Materia Medica, and Tianjin Key Laboratory of Therapeutic Substance of Traditional Chinese Medicine, Tianjin University of Traditional Chinese Medicine, Tianjin, 301617, China. ligen0725@163.com

Yu Jiang: State Key Laboratory of Microbial Technology, Shandong University, Qingdao 266000, P. R. China. [jiangyu950218@163.com](mailto:jiangyu950218@163.com)

Xue Wang: State Key Laboratory of Microbial Technology, Shandong University, Qingdao 266000, P. R. China. [wangxue1118@mail.sdu.edu.cn](mailto:wangxue1118@mail.sdu.edu.cn)

Shi-Jie Cao: State Key Laboratory of Component-based Chinese Medicine, Tianjin University of Traditional Chinese Medicine, Tianjin, 301617, China. [shijiecao0421@hotmail.com](mailto:shijiecao0421@hotmail.com)

Li-Qin Ding: State Key Laboratory of Component-based Chinese Medicine, Tianjin University of Traditional Chinese Medicine, Tianjin, 301617, China. [ruby70303@163.com](mailto:ruby70303@163.com)

Shuang-Yu Bi: State Key Laboratory of Microbial Technology, Shandong University, Qingdao 266000, P. R. China. [shuangyubi@sdu.edu.cn](mailto:shuangyubi@sdu.edu.cn)

Feng Qiu: School of Chinese Materia Medica, Tianjin Key Laboratory of Therapeutic Substance of Traditional Chinese Medicine, and State Key Laboratory of Component-based Chinese Medicine, Tianjin University of Traditional Chinese Medicine, Tianjin, 301617, China. [fengqiu20070118@163.com](mailto:fengqiu20070118@163.com)

Shuang-Jiang Liu: State Key Laboratory of Microbial Technology, Shandong University, Qingdao 266000, China; State Key Laboratory of Microbial Resources, and Environmental Microbiology Research Center (EMRC), Institute of Microbiology, Chinese Academy of Sciences, Beijing 100101, China; University of Chinese Academy of Sciences, Beijing 100049, China. [liusj@sdu.edu.cn](mailto:liusj@sdu.edu.cn)

Chang Liu: State Key Laboratory of Microbial Technology, Shandong University, Qingdao 266000, P. R. China. [liu.c@sdu.edu.cn](mailto:liu.c@sdu.edu.cn)

*Corresponding author: Feng Qiu ([fengqiu20070118@163.com](mailto:fengqiu20070118@163.com)), Shuang-Jiang Liu ([liusj@sdu.edu.cn](mailto:liusj@sdu.edu.cn)), and Chang Liu ([liu.c@sdu.edu.cn](mailto:liu.c@sdu.edu.cn)).


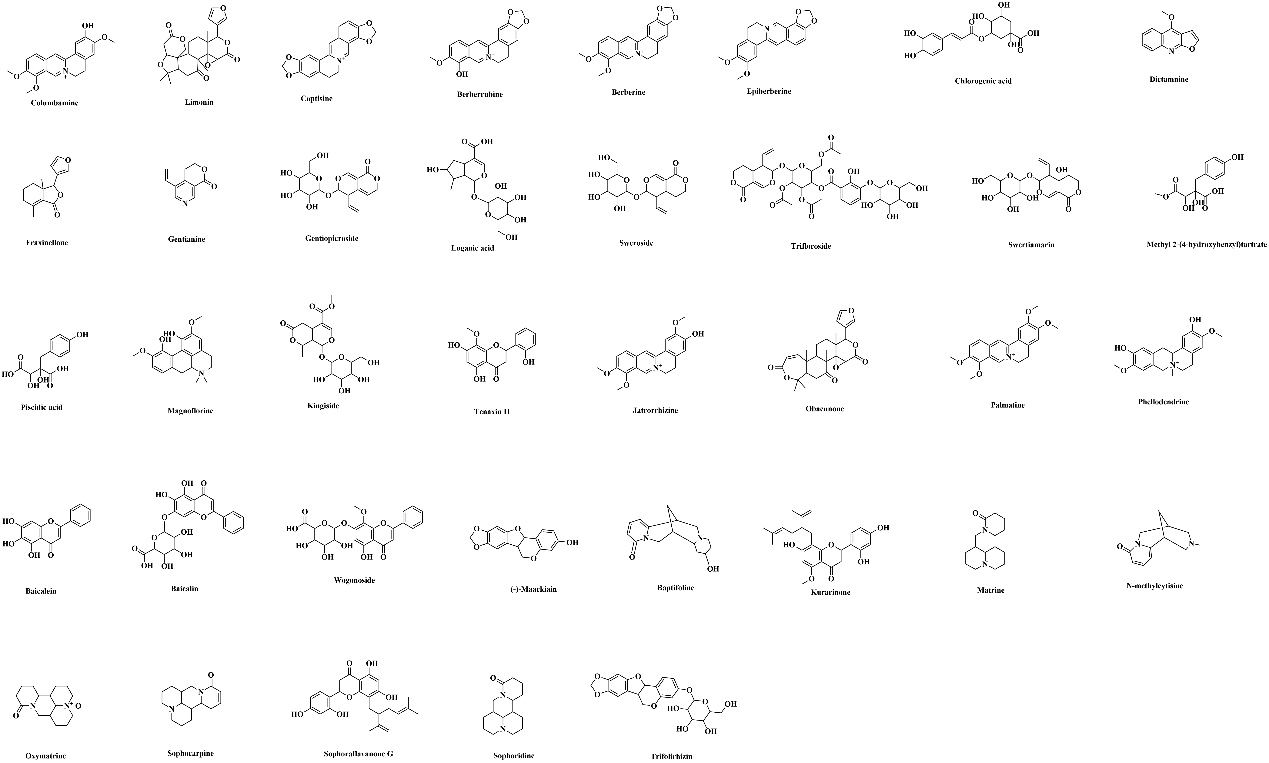


Fig. S1: Chemical structures of 37 pure compounds.


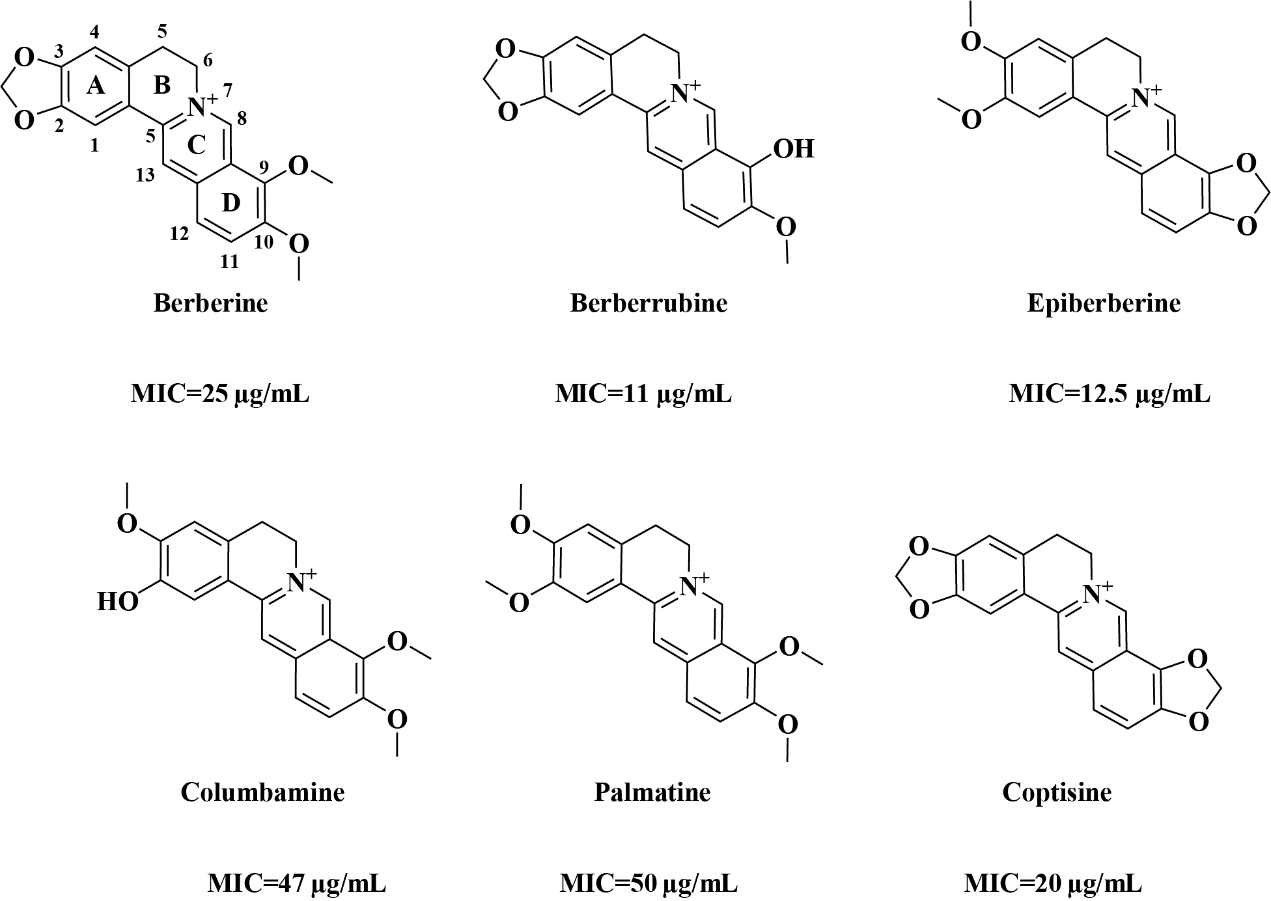


Fig. S2: Chemical structures and minimum inhibitory concentrations (MICs) of berberine derivatives used in the structure–activity relationship (SAR) analysis.


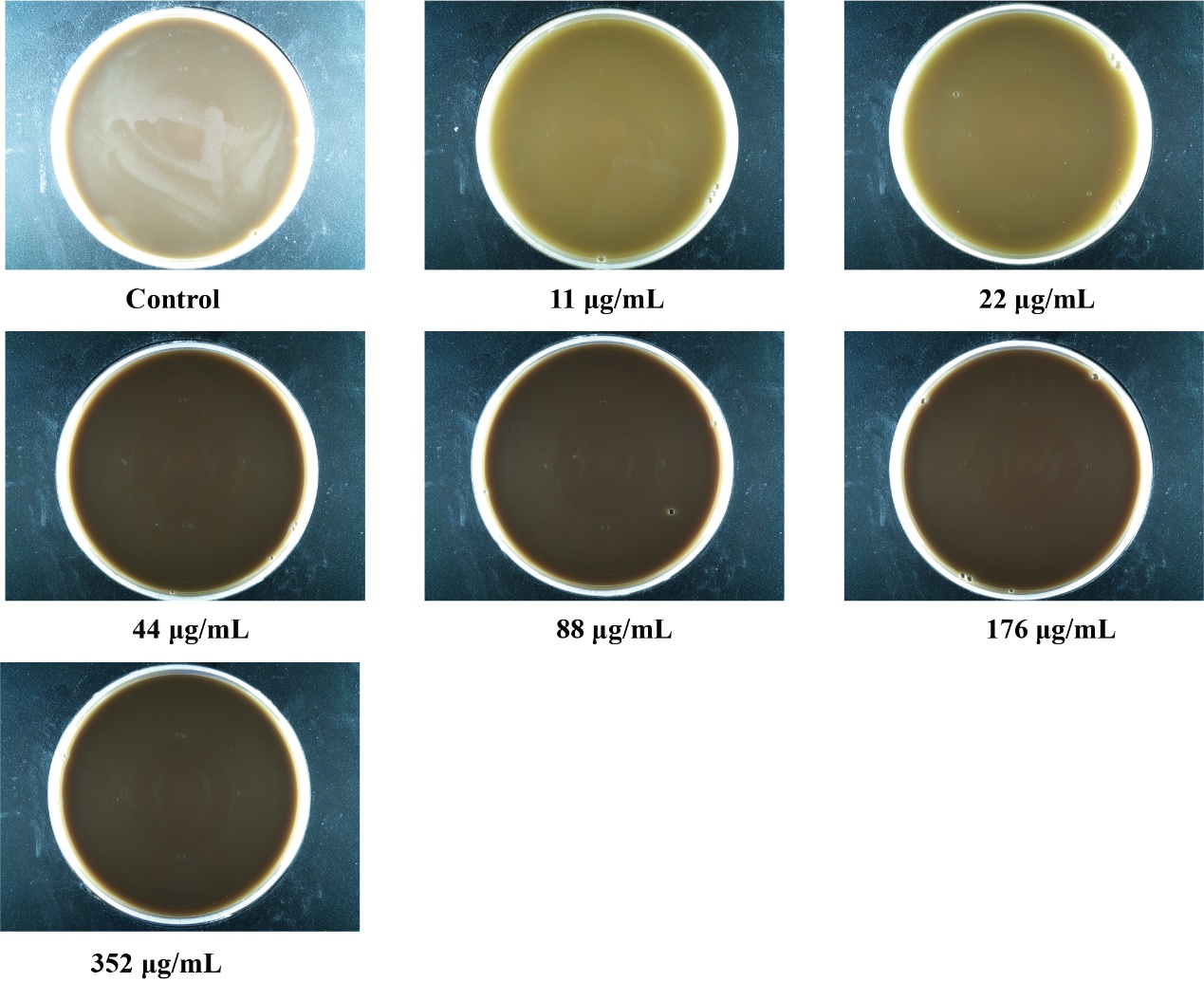


Fig. S3: Representative colony morphology of *Helicobacter pylori* grown under different concentrations of berberrubine.


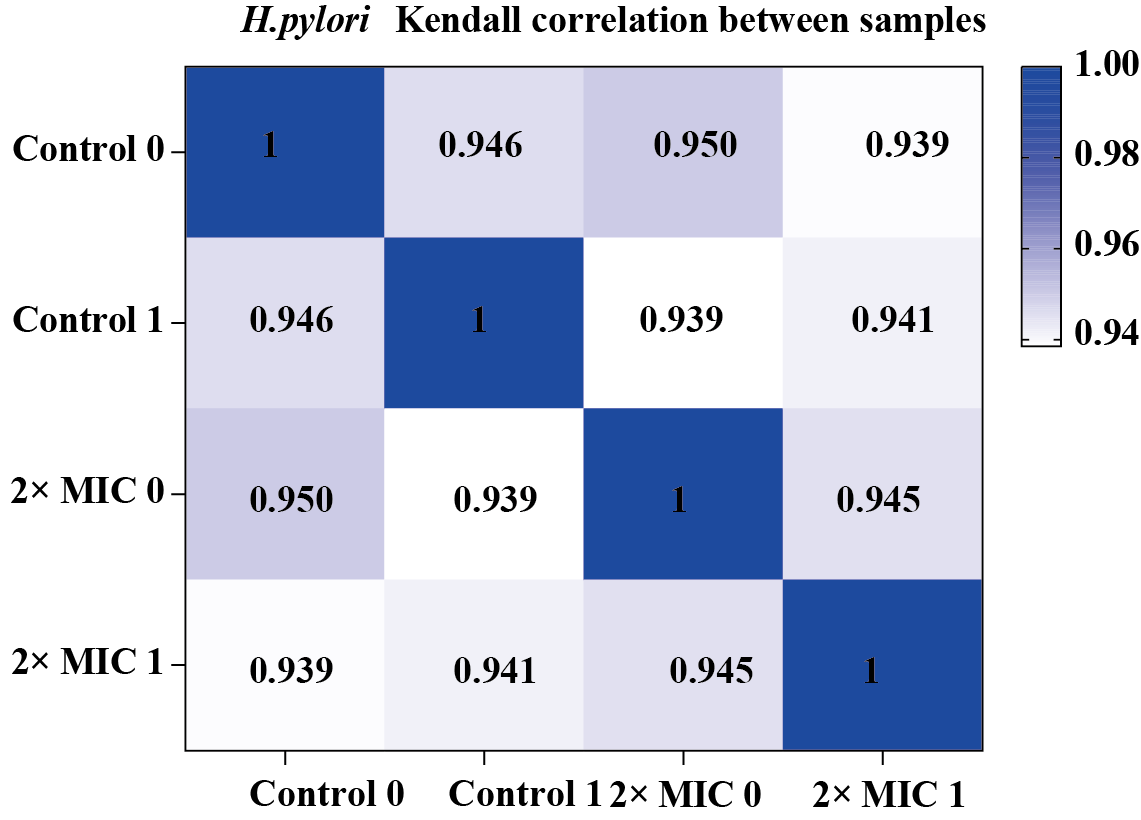


Fig. S4: Kendall correlation coefficient analysis comparing global gene expression profiles between the Control and 2× MIC groups.


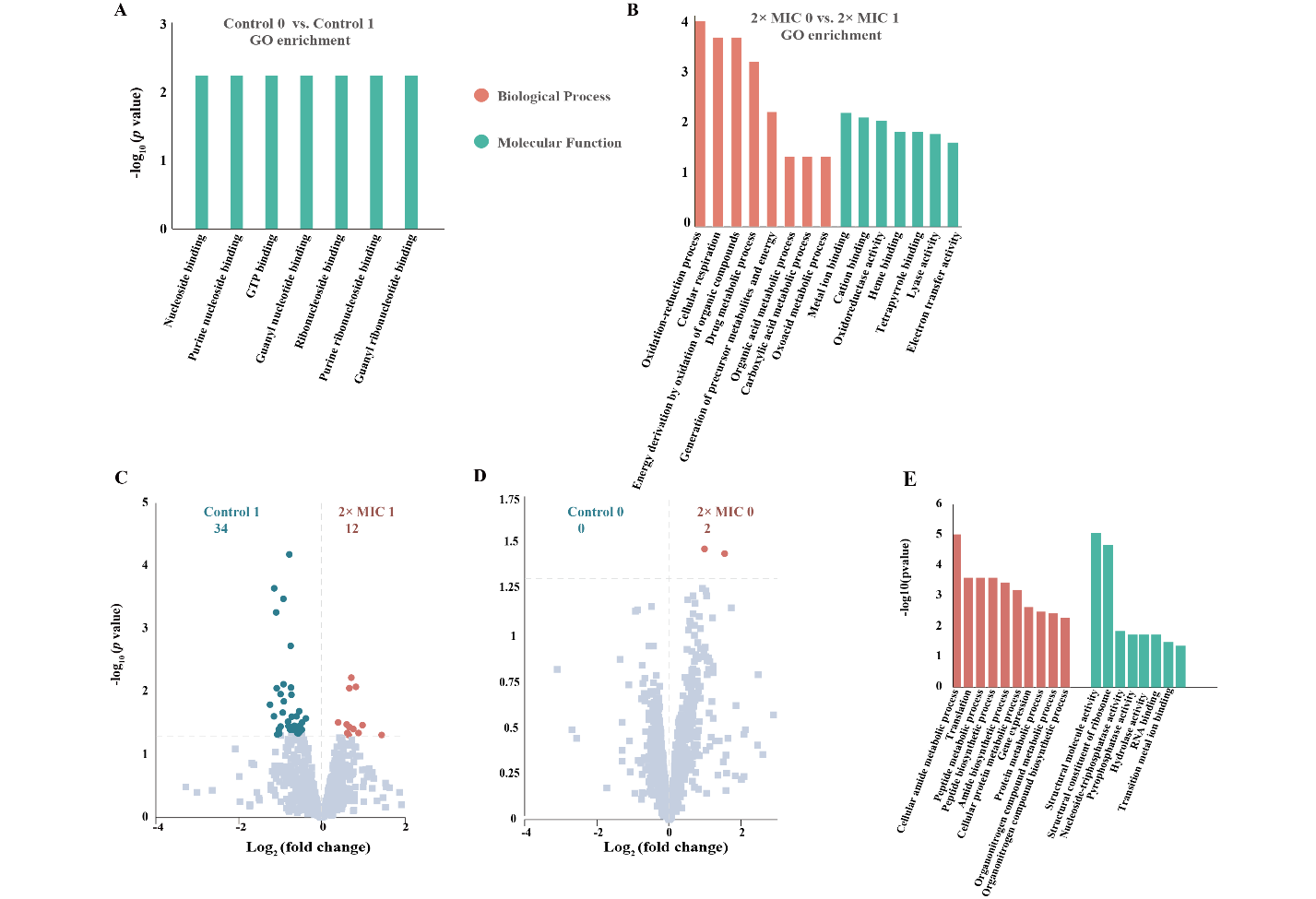


Fig. S5: GO enrichment analysis and volcano plot of DEGs identified in 2 groups.

(A) GO enrichment analysis of DEGs identified in Control 0 vs. Control 1.

(B) GO enrichment analysis of DEGs identified in 2× MIC 0 vs. 2× MIC 1.

(C) Volcano plot of DEGs for Control 1 vs. 2× MIC 1, with |Log_2_ FC| > 0 and *p* value < 0.05.

(D) Volcano plot of DEGs for Control 0 vs. 2× MIC 0, with |Log_2_ FC | > 0 and *p* value < 0.05.

(E) GO enrichment analysis of DEGs identified in 2× MIC 1 vs. Control 1.


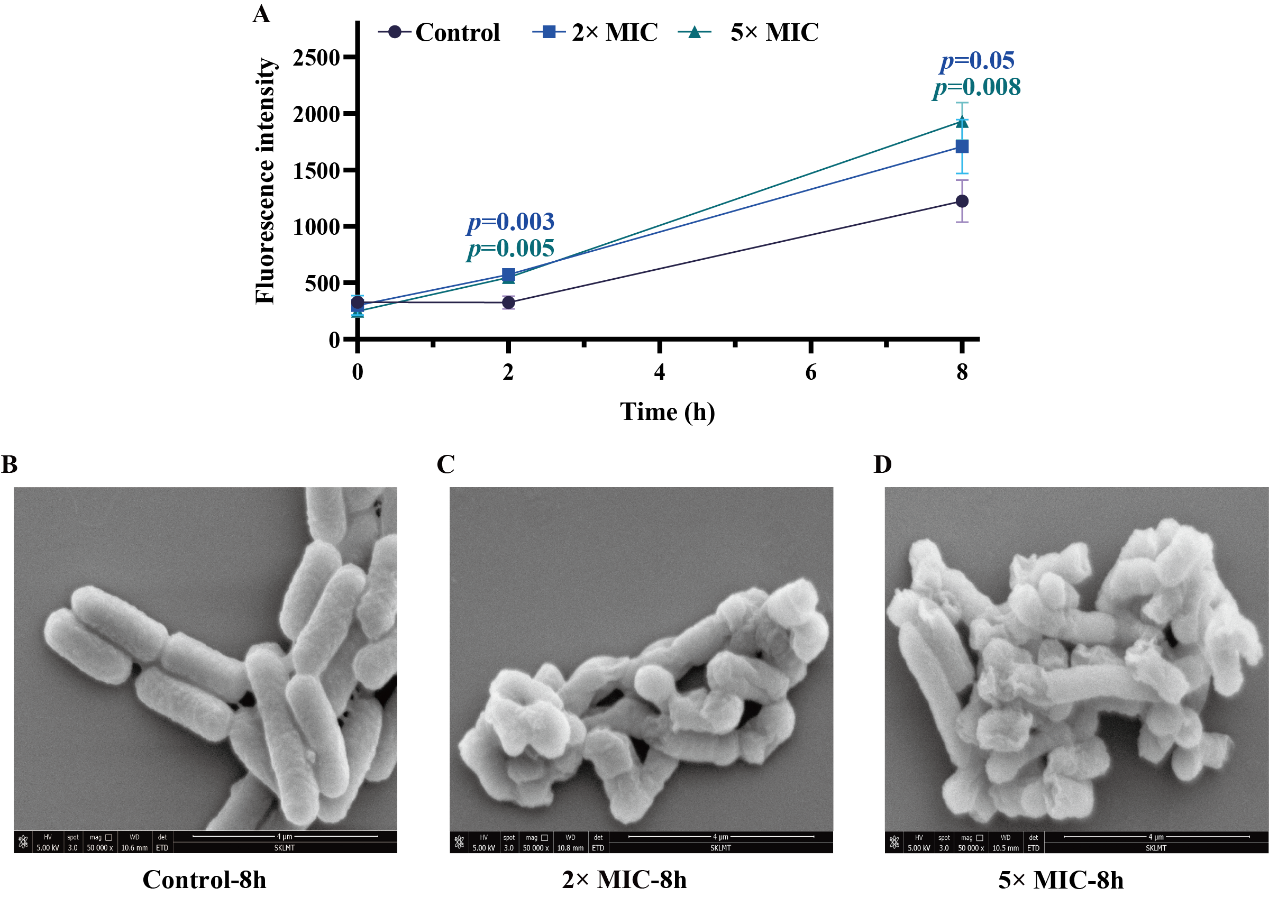


Fig. S6: ROS generation, membrane morphology, and gene expression in *E. coli* following berberrubine treatment

(A) Quantification of intracellular ROS levels in *E. coli* following treatment with vehicle or berberrubine (2× and 5× MIC).

(B-D) SEM images showing cell morphology after 8  h cultivation: (B) untreated Control group, (C) 2× MIC treatment, and (D) 5× MIC treatment.

Data were shown as the mean±S.E.M. The *p* value was calculated by Student's t-test. The *p* value was calculated by Student's t-test and labeled at the corresponding data point.
