# Supplementary material for: Berberrubine inhibits Helicobacter pylori by inducing oxidative stress and impairing membrane integrity
Source: mLife. 2026 Feb 19;5(1):126–30. doi: 10.1002/mlf2.70061 (PMC12948479; doi:10.1002/mlf2.70061)
Supplement: Supplementary file 2 — Supplementary method. [file MLF2-5-126-s003.docx]

**Berberrubine inhibits *Helicobacter pylori* by inducing oxidative stress and impairing membrane integrity**

Min-Zhi Jiang, Chang-Yu Wang, Kai Wang, Xin-Chi Feng, Gen Li, Yu Jiang, Xue Wang, Shi-Jie Cao, Li-Qin Ding, Shuang-Yu Bi, Feng Qiu*, Shuang-Jiang Liu*, Chang Liu*

Min-Zhi Jiang: State Key Laboratory of Microbial Technology, Shandong University, Qingdao 266000, P. R. China. [jiangminzhi2006@126.com](mailto:jiangminzhi2006@126.com)

Chang-Yu Wang: Division of Life Sciences and Medicine, University of Science and Technology of China, 230000, P. R. China. [wcy0817@mail.ustc.edu.cn](mailto:wcy0817@mail.ustc.edu.cn)

Kai Wang: School of Chinese Materia Medica, and Tianjin Key Laboratory of Therapeutic Substance of Traditional Chinese Medicine, Tianjin University of Traditional Chinese Medicine, Tianjin, 301617, China. [kaiwang_2009@163.com](mailto:kaiwang_2009@163.com)

Xin-Chi Feng: School of Chinese Materia Medica, and Tianjin Key Laboratory of Therapeutic Substance of Traditional Chinese Medicine, Tianjin University of Traditional Chinese Medicine, Tianjin, 301617, China. [xiaochi0211@163.com](mailto:xiaochi0211@163.com)

Gen Li: School of Chinese Materia Medica, and Tianjin Key Laboratory of Therapeutic Substance of Traditional Chinese Medicine, Tianjin University of Traditional Chinese Medicine, Tianjin, 301617, China. ligen0725@163.com

Yu Jiang: State Key Laboratory of Microbial Technology, Shandong University, Qingdao 266000, P. R. China. [jiangyu950218@163.com](mailto:jiangyu950218@163.com)

Xue Wang: State Key Laboratory of Microbial Technology, Shandong University, Qingdao 266000, P. R. China. [wangxue1118@mail.sdu.edu.cn](mailto:wangxue1118@mail.sdu.edu.cn)

Shi-Jie Cao: State Key Laboratory of Component-based Chinese Medicine, Tianjin University of Traditional Chinese Medicine, Tianjin, 301617, China. [shijiecao0421@hotmail.com](mailto:shijiecao0421@hotmail.com)

Li-Qin Ding: State Key Laboratory of Component-based Chinese Medicine, Tianjin University of Traditional Chinese Medicine, Tianjin, 301617, China. [ruby70303@163.com](mailto:ruby70303@163.com)

Shuang-Yu Bi: State Key Laboratory of Microbial Technology, Shandong University, Qingdao 266000, P. R. China. [shuangyubi@sdu.edu.cn](mailto:shuangyubi@sdu.edu.cn)

Feng Qiu: School of Chinese Materia Medica, Tianjin Key Laboratory of Therapeutic Substance of Traditional Chinese Medicine, and State Key Laboratory of Component-based Chinese Medicine, Tianjin University of Traditional Chinese Medicine, Tianjin, 301617, China. [fengqiu20070118@163.com](mailto:fengqiu20070118@163.com)

Shuang-Jiang Liu: State Key Laboratory of Microbial Technology, Shandong University, Qingdao 266000, China; State Key Laboratory of Microbial Resources, and Environmental Microbiology Research Center (EMRC), Institute of Microbiology, Chinese Academy of Sciences, Beijing 100101, China; University of Chinese Academy of Sciences, Beijing 100049, China. [liusj@sdu.edu.cn](mailto:liusj@sdu.edu.cn)

Chang Liu: State Key Laboratory of Microbial Technology, Shandong University, Qingdao 266000, P. R. China. [liu.c@sdu.edu.cn](mailto:liu.c@sdu.edu.cn)

*Corresponding author: Feng Qiu ([fengqiu20070118@163.com](mailto:fengqiu20070118@163.com)), Shuang-Jiang Liu ([liusj@sdu.edu.cn](mailto:liusj@sdu.edu.cn)), and Chang Liu ([liu.c@sdu.edu.cn](mailto:liu.c@sdu.edu.cn)).

## Materials and methods

### Natural components preparation

The ethanol and water extracts of several medicinal plants were prepared and analyzed in our laboratory. *Gentiana scabra* Bge. water extract was prepared by soaking the dried roots and rhizomes overnight, followed by reflux extraction three times with 10-fold distilled water (w/v) for 3 h each. The combined extracts were concentrated using rotary evaporation at 60°C and freeze-dried, yielding a crude extract with a 53.5% extraction rate. In addition, the ethanol extract of *Gentiana scabra* Bge. was prepared, and the following compounds were isolated and identified: gentiopicroside, trifloroside, swertiamarin, sweroside, loganic acid, kingiside, and gentianine.

For *Sophora flavescens* Aiton, the dried roots were first soaked for 30 min and subjected to reflux extraction twice with 30-fold distilled water (w/v) for 20 min each time, yielding a water extract with a 25% extraction rate after concentration and freeze-drying. The dried roots were also extracted five times with 3-fold 70% ethanol (w/v) for 1.5 h each time, and the ethanol extract was obtained with a 26% yield after concentration and freeze-drying. The following compounds were isolated and identified: matrine, oxymatrine, sophocarpine, sophoridine, baptifoline, N-methylcytisine, trifolirhizin, kurarinone, (-)-Maackiain, sophoraflavanone G, piscidic acid, and methyl punicate.

For *Dictamnus dasycarpus* Turcz., the dried root barks were pulverized and extracted twice with 10-fold distilled water (w/v) for 2 h each time. The combined extracts were concentrated and freeze-dried, yielding a water extract with a 21.9% extraction rate. The ethanol extract of *Dictamnus dasycarpus* Turcz. was obtained similarly, with a yield of 20.8%. Fraxinellone, limonin, and dictamnine were isolated and identified.

For berberrubine, in a round-bottom flask, 1.01g of berberine hydrochloride and 15ml of N,N-Dimethylformamide (DMF) were added, along with a few boiling stones. The mixture was refluxed under condensation at 190°C for 20 min. Then the reaction flask was removed and while hot, 1.5 times the amount of water was added to dilute and to cool the solution. The solution was refrigerated overnight to allow complete crystallization. Finally, perform suction filtration and dry to obtain 0.7747g of red powdery solid - berberrubine, with a yield of 76.7%. Through further purification, berberrubine with a purity of > 95% was obtained.

Lastly, the dried bark of *Phellodendron chinense* C.K.Schneid. was ground into powder, soaked for 30 min in ten volumes of distilled water (w/v), and boiled twice for 2 h per time. The combined filtrates were concentrated and freeze-dried, yielding a water extract with a final extraction rate of 19.2%. Palmatine, jatrorrhizine, obacunone, phellodendrine, magnoflorine, and chlorogenic acid were isolated and identified. *Coptis chinensis* Franch and *Scutellaria baicalensis* Georgi extracts were prepared in our laboratory following previous studies ^1, 2^. All compounds were identified using HPLC with purities >95%. *Gentiana scabra* Bge., *Sophora flavescens* Aiton, *Dictamnus dasycarpus* Turcz., *Phellodendron chinense* C.K.Schneid., *Coptis chinensis* Franch, and *Scutellaria baicalensis* Georgi were validated by Professor Lijuan Zhang of Tianjin University of Traditional Chinese Medicine.

### Strains, reagents, and media

The bacterial strains used in this study were H. pylori and E. coli, which were stored in our laboratory. H. pylori was cultured in modified Brain Heart Infusion (BHI) medium, which was supplemented with 3.5 g/L yeast extract, 17.5 g/L peptone, and 10% fetal bovine serum (v/v), while E. coli was cultured in MH broth medium. Both BHI and MH broths were purchased from Hopebio, Qingdao, China. 46 natural products were obtained from Tianjin University of Traditional Chinese Medicine, Tianjin, China. The powders were dissolved in 100 μL dimethyl sulfoxide (DMSO) and then diluted with 900 μL of deionized water (ddH₂O) to a final volume of 1 mL.

### Antimicrobial activity test

The minimum inhibitory concentrations (MICs) of 46 natural products against the tested strains were determined following the broth microdilution method recommended by the Clinical and Laboratory Standards Institute (CLSI) ^3^. The starting concentrations of the natural products are listed in Table S4, and each compound was subjected to 11 serial dilutions. PBS served as the positive control, with all assays performed in triplicate. The 46 natural products were serially diluted 2-fold in BHI or MH broth in sterile 96-well plates. Each well was then inoculated with 100 μL of bacterial suspension at a concentration of 1 × 10^10^ cells/mL. After co-incubation at 37 °C for 48 h (for H. pylori) or 24 h (for E. coli), the MIC was defined as the lowest concentration at which no noticeable turbidity was observed, by comparing the drug concentration to that in the control wells.

**Determination of the MBC of berberrubine.**

Berberrubine was serially diluted (MIC, 2×, 4×, 8×, 16×, and 32× MIC) in modified BHI medium supplemented with 10% fetal bovine serum and dispensed into 96-well plates. Log-phase H. pylori was adjusted to 1 × 10^10^ CFU/mL and inoculated into each well. Cultures were incubated at 37 ℃ under microaerophilic conditions for 48 h, after which 100 μL from each well was spread onto BHI agar and incubated for an additional 48 h. Colony formation was recorded, and the lowest concentration preventing visible growth was defined as the MBC. Based on standard criteria, agents with an MBC/MIC ratio < 4 were classified as bactericidal, whereas those with ratios of 4-32 were considered bacteriostatic ^4^.

### Transcriptomic sample preparation

Based on the previously determined sublethal concentrations of berberrubine against H. pylori, 2 sets of samples were selected for transcriptomic analyses to explore the antibacterial mechanisms of berberrubine. The bacteria were inoculated with 1% (v/v), cultured at 37°C until the OD_600_ = 0.6, and then treated as follows: 1) Control: sterile modified BHI without berberrubine; 2) 2× MIC : sterile modified BHI with 1/2 MIC berberrubine, and then incubated at 37°C. The harvested cells were collected after being exposed to 1/2 MIC berberrubine for 2 h. A sub-inhibitory concentration was selected to minimize nonspecific stress responses, while the 2 h duration—shorter than the estimated doubling time (~2.7 h)—ensured transcriptomic profiling within a single replication cycle, avoiding secondary effects from cell death or growth phase transitions. The cell suspensions were centrifuged at 10,000 rpm for 2 min to remove the culture medium. The cell pellets were immediately frozen with liquid nitrogen and temporarily stored at -80°C before the following RNA extraction.

### Transcriptomic data generation and analysis

Total RNA was extracted using TRIzol reagent (Vazyme, China) for RNA-seq. Raw reads in fastq format were processed with in-house Perl scripts to remove adapter sequences, poly-N reads, and low-quality reads, generating high-quality clean data. Read quality was assessed by calculating Q20, Q30, and GC content. The reference genome and annotation files (Ref. GCA_007836855.1) were downloaded from NCBI, and the genome index was constructed. Clean paired-end reads were aligned to the reference genome using Hisat2 v2.0.5 ^5^ , and gene-level read counts were obtained with FeatureCounts v1.5.0-p3. Expression levels were normalized as FPKM values. Differential expression analysis was performed using DESeq2 v1.20.0, with significant DEGs defined as p < 0.05 and |log2FC| > 0. Functional enrichment of DEGs was conducted using Gene Ontology (GO), with significantly enriched terms identified at p < 0.05.

### Detection of ROS generation

Cells were centrifuged at 10,000 rpm for 2 min, and the pellets were resuspended to an OD_600_ of 1.0. The suspensions were incubated with 10 μM 2′,7′-dichlorofluorescein diacetate (DCFH-DA) in the dark for 30 min. Excess dye was removed by centrifugation and two washes with PBS. Fluorescence was then measured at 488 nm (excitation) and 525 nm (emission) using a multifunctional microplate reader.

### Assessment of ROS involvement in the bacteriostatic effect of berberrubine

To evaluate whether ROS contribute to the bacteriostatic activity of berberrubine, a growth assay was conducted using *H. pylori* cultured under three different conditions: (1) vehicle control, (2) 5× MIC berberrubine, and (3) 5× MIC berberrubine co-treated with 20 mM N-acetylcysteine (NAC), an antioxidant previously reported to neutralize intracellular ROS ^6^. A seed culture of *H. pylori* (1 × 10¹⁰ CFU/mL) was inoculated at 1% (v/v) into modified BHI medium supplemented with 10% fetal bovine serum for each treatment group. Cultures were incubated under microaerophilic conditions at 37 °C, and bacterial growth was monitored by measuring OD_600_ at 2 h intervals over the incubation period to generate growth curves.

### Scanning electron microscope (SEM)

Two milliliters of bacterial culture were centrifuged at 3,000 rpm for 5 min, and the pellets were fixed in 2.5% glutaraldehyde at 4 °C for 4 h. The samples were washed three times with PBS and dehydrated through a graded ethanol series (30%, 50%, 70%, 85%, and 95% once each, and 100% twice), with each step lasting 15-20 min. Finally, the samples were replaced with tert-butyl alcohol twice, 20 min each time, before critical point drying. After dehydration and tert-butyl alcohol replacement, the samples were freeze-dried overnight at -20 °C. The dried samples were mounted on SEM stubs, coated with a thin layer of gold by sputtering, and observed under a scanning electron microscope (Quanta 250 FEG, FEI, USA) at 50,000× magnification.

### Assessment of berberrubine’s effect on cell membrane integrity

To assess the impact of berberrubine on *H. pylori* membrane integrity, 1 × 10¹⁰ CFU/mL cells suspended in BHI media were treated with either 5× MIC berberrubine alone or in combination with 20 mM N-acetylcysteine (NAC). Following a 2 h incubation at 37 °C, cell membrane integrity and viability were evaluated using the Live/Dead BacLight Bacterial Viability Kit (Thermo Fisher Scientific), following the manufacturer’s protocol**. Imagines were generated by fluorescent microscopy (NIKON Ti-E, Nikon, Japan) and SEM.**

### Statistical analysis

All data were analyzed using GraphPad Prism 10 software and presented as the mean ± S.E.M. Figures were visualized using Adobe Illustrator. Data comparisons were performed using Student's t-test.

### Supplementary References

1. Feng X, Wang K, Hu X, Chai L, Cao S, Ding L, et al.Systematic screening and characterization of absorbed constituents and in vivo metabolites in rats after oral administration of *Rhizoma coptidis* using UPLC-Q-TOF/MS. *Biomed Chromatogr.* 2020; 34: e4919.

2. Liu ZB, Sun CP, Xu JX, Morisseau C, Hammock BD, Qiu F. Phytochemical constituents from Scutellaria baicalensis in soluble epoxide hydrolase inhibition: kinetics and interaction mechanism merged with simulations. *Int J Biol Macromol.* 2019; 133: 1187-1193.

3. Humphries R, Bobenchik AM, Hindler JA, Schuetz AN. Overview of changes to the clinical and laboratory standards institute *performance standards for antimicrobial susceptibility testing*, M100, 31st Edition. *J Clin Microbiol.* 2021; 59: e0021321.

4. Kim HW, Lee JW, Yu AR, Yoon HS, Kang M, Lee BS, et al. Isoegomaketone exhibits potential as a new *Mycobacterium abscessus* inhibitor. *Front Microbiol.* 2024; 15: 1344914.

5.She PF, Liu YQ, Xu LL, Li ZH, Li YM, Liu SS, et al. L007-0069 kills in high resistant phenotypes. *Cell Mol Life Sci.* 2022; 79: 552.

6. Feng Q, Hu K, Hu H, Lu Y, Zhang H, Wang G, et al. Berberine derivative DCZ0358 induce oxidative damage by ROS-mediated JNK signaling in DLBCL cells. *Int Immunopharmacol.* 2023; 125: 111139.
